# Evidence for Allele-Specific Levels of Enhanced Susceptibility of Wheat *mlo* Mutants to the Hemibiotrophic Fungal Pathogen *Magnaporthe oryzae* pv. *Triticum*

**DOI:** 10.3390/genes11050517

**Published:** 2020-05-07

**Authors:** Katrin Gruner, Tobias Esser, Johanna Acevedo-Garcia, Matthias Freh, Michael Habig, Roxana Strugala, Eva Stukenbrock, Ulrich Schaffrath, Ralph Panstruga

**Affiliations:** 1 Unit of Plant Molecular Cell Biology, Institute for Biology I, RWTH Aachen University, Worringerweg 1, 52056 Aachen, Germany; Katrin.Gruner@rwth-aachen.de (K.G.); johanna.acevedo-garcia@keygene.com (J.A.-G.); matthias.freh@rwth-aachen.de (M.F.); 2 Department of Plant Physiology, Institute for Biology III, RWTH Aachen, Worringerweg 1, 52056 Aachen, Germany; tobias.esser2@rwth-aachen.de (T.E.); roxana.strugala@rwth-aachen.de (R.S.); schaffrath@bio3.rwth-aachen.de (U.S.); 3 Department of Environmental Genomics, Christian-Albrechts University of Kiel, Am Botanischen Garten 1–9, 24118 Kiel, Germany; mhabig@bot.uni-kiel.de (M.H.); estukenbrock@bot.uni-kiel.de (E.S.); 4 Environmental Genomics Group, Max Planck Institute for Evolutionary Biology, August-Thienemann-Straße 2, 24306 Plön, Germany

**Keywords:** *Blumeria graminis*, hexaploid bread wheat, *Magnaporthe oryzae*, *Mlo*, plant disease resistance, powdery mildew, TALEN, TILLING, *Zymoseptoria tritici*

## Abstract

Barley *mlo* mutants are well known for their profound resistance against powdery mildew disease. Recently, *mlo* mutant plants were generated in hexaploid bread wheat (*Triticum aestivum*) with the help of transgenic (transcription-activator-like nuclease, TALEN) and non-transgenic (targeted induced local lesions in genomes, TILLING) biotechnological approaches. While full-gene knockouts in the three wheat *Mlo* (*TaMlo*) homoeologs, created via TALEN, confer full resistance to the wheat powdery mildew pathogen (*Blumeria graminis* f.sp. *tritici*), the currently available TILLING-derived *Tamlo* missense mutants provide only partial protection against powdery mildew attack. Here, we studied the infection phenotypes of TALEN- and TILLING-derived *Tamlo* plants to the two hemibiotrophic pathogens *Zymoseptoria tritici*, causing Septoria leaf blotch in wheat, and *Magnaporthe oryzae* pv. *Triticum* (*MoT*), the causal agent of wheat blast disease. While *Tamlo* plants showed unaltered outcomes upon challenge with *Z. tritici*, we found evidence for allele-specific levels of enhanced susceptibility to *MoT*, with stronger powdery mildew resistance correlated with more invasive growth by the blast pathogen. Surprisingly, unlike barley *mlo* mutants, young wheat *mlo* mutant plants do not show undesired pleiotropic phenotypes such as spontaneous callose deposits in leaf mesophyll cells or signs of early leaf senescence. In conclusion, our study provides evidence for allele-specific levels of enhanced susceptibility of *Tamlo* plants to the hemibiotrophic wheat pathogen *MoT*.

## 1. Introduction

Bread wheat (*Triticum aestivum*) is one of the world´s most important food crop species [1]. Like other cereals, it can suffer from a variety of microbial diseases. Common practices for disease control comprise the deployment of resistant cultivars and the application of agrochemicals (pesticides). However, genetically conditioned disease resistance, which is highly desired from the perspective of costs, practicability and sustainability, is often ephemeral due to the rapid evolution of pathogenic microbes that can overcome plant immunity [2]. 

Powdery mildew is a common and widespread disease of angiosperm plants that is caused by ascomycete fungi [3]. These fungi thrive on the basis of an obligate biotrophic lifestyle, i.e., they require living host tissue for growth and reproduction [4]. Powdery mildew also affects important food crops such as wheat and barley. The causal agent of the disease on these cereals is *Blumeria graminis*—a species that exists in various highly host-specialized variants (*formae speciales*; [5]) and has been elected one of the top 10 fungal pathogens in terms of scientific and economic importance [6].

Resistance of cereals to powdery mildew is often conditioned by dominantly inherited resistance (*R*) genes, which usually confer race-specific immunity [7,8]. Unlike prototypical *R* genes, recessively inherited loss-of-function *mlo* (*mildew locus o*) mutations give rise to race non-specific powdery mildew resistance [9]. Originally discovered as a natural mutant of barley [10], *mlo*-mediated resistance has been recently shown to represent a common phenomenon in angiosperm plants (reviewed by [11]). Typically, *mlo* resistance can be induced by loss-of-function mutations in a single *Mlo* gene, although exceptions exist such as in *Arabidopsis thaliana* where mutations in three *Mlo* genes contribute to resistance via unequal genetic redundancy [12]. Barley *mlo* mutants have been extensively used in European agriculture and were found to confer stable, effective and long-lasting resistance [13,14]. The molecular basis of this type of disease resistance is still incompletely understood [11,15].

In hexaploid bread wheat, three orthologs of the barley *Mlo* gene (*TaMlo* homoeologs) are present in the A, B and D genome [16]. Previous transgenic and non-transgenic approaches led to wheat mutants with genetic lesions in all three *TaMlo* homoeologs [17,18]. The transgenic approach, mediated by transcription activator-like effector nucleases (TALEN), resulted in knockout mutants with apparently complete resistance to the wheat powdery mildew pathogen, *Blumeria graminis* f.sp. *tritici* (*Bgt*) [17]. By contrast, the non-transgenic approach, achieved via targeted induced local lesions in genomes (TILLING) technology resulting in missense mutations in the *TaMlo* homoeologs, yielded mutants with residual activity and accordingly partial *Bgt* resistance [18]. Recently, TILLING-derived *mlo* mutants were also reported in tetraploid durum wheat (*Triticum turgidum* [19]).

In some species (such as barley and *Arabidopsis thaliana*), powdery mildew-resistant *mlo* mutants show pleiotropic phenotypes such as the spontaneous formation of callose-containing cell wall appositions and signs of early leaf senescence [12,20,21,22,23]. These phenotypes are variable and their occurrence and severity appear to depend on yet poorly characterized environmental conditions. Interestingly, powdery mildew-resistant *mlo* mutants in some plant species, such as tomato and pea, seem to lack such pleiotropic phenotypes [24,25].

Apart from powdery mildew, for which mutations in *Mlo* genes cause durable broad-spectrum resistance, *mlo* mutants modulate the outcome of interactions with some other pathogens as well. The first report in this respect described the enhanced susceptibility of barley *mlo* mutants to the rice blast fungus *Magnaporthe oryzae*, which is also a pathogen of barley [26]. *M. oryzae* recently emerged as a novel pathogen of wheat in Brazil, likely enabled by the extensive use of a particular wheat cultivar, which permitted a host jump as a consequence of the loss of a crucial avirulence determinant in the fungus [27]. The wheat blast disease is an upcoming threat for wheat cultivation and, thereby, for global food security [28]. The disease was first reported in 1985 for South America and spread to Asia in 2016 [29]. There is an ongoing debate whether or not isolates causing blast disease on wheat represent a novel species or a particular lineage within the *M. oryzae* species complex [30,31]. For this study, we will follow the latter perception and refer to the pathogen as *M. oryzae* pathotype *Triticum* (*MoT*).

Here, we analyzed a set of bread wheat *Tamlo* mutants, including transgenic TALEN and non-transgenic TILLING mutants, to assess their disease phenotypes against fungal phytopathogens with different lifestyles. We focused on powdery mildew (*Bgt*), and the two hemibiotrophic pathogens *MoT* and *Zymoseptoria tritici*. We further assessed the potential of the *Tamlo* mutants to develop undesired pleiotropic phenotypes by histochemically analyzing the formation of spontaneous callose deposits and scoring signs of early leaf senescence. Results of our experiments suggest that similar to barley *mlo* mutants, strong *Tamlo* mutants seem to be prone to enhanced *MoT* susceptibility.

## 2. Materials and Methods

### 2.1. Plant Material and Growth Conditions

The hexaploid bread wheat cultivar (cv.) Cadenza (a British spring wheat variety) and its derived TILLING *Tamlo* lines as well as the Chinese cv. Kenong 199 (KN199, a winter wheat variety) and its derived TALEN *Tamlo* triple-mutant lines were described before [17,18]. Wheat plants were grown and propagated as reported previously [18] except for the used soil type, which was changed to SoMi513 (HAWITA, Vechta, Germany). Barley cv. Ingrid and its isogenic null mutant backcross Ingrid (BCI) *mlo*-3 have been described before [32]. The BCI *mlo*-3 line was generated by seven backcrosses of the original γ-ray-mediated mutant in the genetic background of cv. Malteria Heda with the recurrent parent cv. Ingrid, followed by at least six selfings [33]. 

### 2.2. Bgt Infection Assays

The German *Bgt* field isolate JA82 was propagated as described before [18] with minor changes. During a later stage in the course of this study, the wheat variety used for fungal propagation was changed to the commercially available spring wheat variety KWS Sharki (https://www.kws.com/de/de/produkte/getreide/weizen/sortenuebersicht/kws-sharki/) and 7-day-old seedlings were used in a weekly propagation routine. The infection assays with *Bgt* were performed as reported previously [18]. In brief, primary leaves of 10-day-old seedlings were fixed on a polycarbonate platform, emerging secondary leaves were cut short, and the primary leaves were equally inoculated with *Bgt* conidiospores. For evaluation of *Bgt* host cell entry rates, inoculated leaves were harvested 3 days later and fixed in a destaining solution. Epiphytic fungal structures on the destained leaves were visualized with Coomassie Brillant Blue R-250 (Carl Roth GmbH, Karlsruhe, Germany) and evaluated under the microscope by scoring at least 200 interaction sites (attacked cells with haustoria/hyphae versus attacked cells with appressoria). Macroscopic evaluation of the leaves was undertaken at 6 days post infection (dpi).

### 2.3. Z. tritici Infection Assays

The Dutch *Z. tritici* isolates IPO323 (local ID: Zt244) is available from the Westerdijk Institute (Utrecht, The Netherlands) with the accession numbers CBS115943. *Z. tritici* strains Zt05 (ID: MgDk09_U34) [34] and Zt153 (ID: Zt_Ger_13_5_1_1) [35] were isolated in Denmark and Germany, respectively, and are available upon request. Strains were maintained on either liquid yeast malt sucrose (YMS) broth (4 g L^−1^ yeast extract, 4 g L^−1^ malt extract, 4 g L^−1^ sucrose) at 18 °C on an orbital shaker or on solid YMS medium (supplemented with 20 g L^−1^ agar) at 18 °C.

Infection phenotypes were determined as described before [36]. Briefly, seeds of the seven tested wheat genotypes (cv. Cadenza, four TILLING *Tamlo* triple-mutant lines, KN199 and the TALEN *Tamlo* triple-mutant line) were germinated on wet Whatman paper for four days before potting. Wheat seedlings were further grown for seven days before inoculation. The *Z. tritici* strains were cultivated on YMS solid medium for 5 days at 18 °C before the cells were scraped from the plate surface. A ~5 cm long section on the secondary leaf of each plant was infected by brushing a cell suspension with 10^7^ cells mL^−1^ in 0.1% Tween 20 on the abaxial and adaxial side of leaf. Plants were placed into sealed bags containing ~1 L of water for 48 h to facilitate infection at maximum air humidity. Plants were grown under constant conditions with a day–night cycle of 16 h light (~200 µmol m^−2^ s^−1^) and 8 h darkness in growth chambers at 20 °C. Infected leaf areas were harvested at 21 dpi, pressed for five days at 4 °C and then scanned using a flatbed scanner (HP Photosmart C4580, HP, Böblingen, Germany). Pycnidia density and percentage of the leaf area covered by lesions were determined using automated image analyses as described previously [36,37].

### 2.4. MoT Infection Assays

Wheat plants were pre-germinated for 24 h on wet filter paper and then germlings were transferred to standard soil (type ED73, Balster Einheitserdewerk GmbH, Froendenberg, Germany). Plastic pots (7 × 7 × 8 cm) with five germlings each were kept in a growth chamber at a 16 h light (200–250 μmol s^−1^ m^−2^)/8 h dark rhythm at 20 °C and 65% relative humidity.

The *Magnaporthe* isolates BR32 [38] and Br116.5 [39] were kindly provided by Didier Tharreau (CIRAD, Montpellier, France) and by Yukio Tosa (Kobe University, Japan), respectively. Inoculations of wheat plants with these isolates were performed as described previously [40,41]. In brief, fungal cultures were maintained on oatmeal agar (20 g L^−1^ agar, 2 g L^−1^ yeast extract, 10 g L^−1^ starch, 30 g L^−1^ oat flakes) at 23 °C in the dark. Sporulation was induced by keeping fungal cultures for two weeks at a 16 h light/8 h dark regime under black light at 22 °C. After two weeks, conidia were harvested by rinsing plates with water and filtering through three layers of gauze. For inoculation, the conidia suspension was initially adjusted to 400,000 spores mL^−1^, then diluted 1:2 with surfactant (2 g L^−1^ gelatin, 1 mL L^−1^ Tween) to a final concentration of 200,000 conidia mL^−1^ and finally spray-inoculated onto plants. After incubation at 24–26 °C and 100% relative humidity for 24 h in the dark, inoculated plants were covered with a plastic hood and kept under the growing conditions described above. Classification of infection sites was performed as described before [42].

### 2.5. Scoring of Spontaneous Callose Deposition

Primary leaves of wheat and barley plants were harvested at a plant age of 31 and 24 days, respectively. In both cases, the upper 3 cm of the leaves were removed, and the following 3 cm sections were analyzed. Barley leaf samples were cleared and fixed in ethanol/chloroform (3:1) with 0.15% (*w*/*v*) trichloroacetic acid as described before [20], while wheat leaf sections were cleared in ethanol/acetic acid (3:1). For staining, all samples were incubated in 0.067 M potassium-phosphate buffer (pH 5.8) with 0.05% (*w*/*v*) aniline blue for at least 12 h (adapted from [20]). Leaf sections were analyzed by epifluorescence microscopy using the Keyence BZ-9000 microscope (DAPI filter set; excitation wavelength: 370 nm, detection at 509 nm) and pictures captured using the BZ-II viewer software with exposure times between 1/2 and 1/9 s as well as high black balance to reduce background fluorescence. Subsequently, micrographs of wheat samples were analyzed using CellProfiler software (version 3.1.9; [43]) for batch analysis, while the barley samples were scored manually due to high background fluorescence of the leaf veins, which interfered with the automated evaluation.

### 2.6. Assessment of Leaf Chlorosis/Necrosis

As for the scoring of spontaneous callose depositions, primary leaves of wheat and barley plants were removed for analysis at a plant age of 31 and 24 days, respectively. Leaves were fixed on a light panel (Kaiser Slimlite, Kaiser Fototechnik GmbH & Co. KG, Buchen, Germany) and pictures of the upper 5 cm of the leaves were taken. The contrast of the photographs was increased to further reduce the background and the pictures were analyzed with the “leaf necrosis classifier” software (https://lnc.proteomics.ceitec.cz/home).

### 2.7. Statistical Analysis of The Data

Statistical analyses and graph generation were performed using the GraphPad Prism software (GraphPad Prism Software Inc., San Diego, CA, USA). In boxplot graphs, the center lines show the medians and upper and lower box limits indicate the 25th and 75th percentiles, respectively, with all data points represented by colored dots. Statistical analyses were performed using either a regular two-way analysis of variance (ANOVA; *p* < 0.05) or a one-way ANOVA test with Tukey’s method for multiple comparisons (multi-paired ANOVA; *p* < 0.05) as indicated. The latter was used to generate significance groups represented as letters above the boxplots in the respective graphs.

## 3. Results

### 3.1. Tamlo Lines Show Allele-Dependent Levels of Powdery Mildew Resistance

We first completed our previously initiated, yet at the time incomplete, collection of four TILLING *Tamlo* triple-mutant lines bearing different allele combinations and thus mutational events in the *TaMlo* homoeologs of the wheat A, B and D genomes ([18] and Figure S1). In addition, we expanded the set of the two triple-mutant lines described before (designated lines 1 and 2; [18]), harboring *Tamlo* alleles with experimentally determined medium to very strong effects, by selecting independent progenies of respective intermutant crosses [18], resulting in the same allele combinations. This led to three additional independent segregants for triple-mutant line 1 and two additional independent segregants for triple-mutant line 2. We further selected two new triple-mutant lines (designated lines 3 and 4; with two and three independent segregants, respectively) that are based on *Tamlo* alleles with experimentally determined weak to strong effects (Figure S1). Therefore, we expected lines 3 and 4 to exhibit lower levels of powdery mildew resistance than observed for lines 1 and 2 [18]. In addition to the four TILLING triple-mutant lines, we identified all corresponding single- and double-mutant combinations related to the four triple-mutant lines. In summary, we retrieved a complete set of single-, double- and triple-mutant combinations of the four TILLING triple-mutant lines (lines 1–4) harboring different *Tamlo* allele combinations with expected differential powdery mildew infection phenotypes [18], and with each of the respective triple-mutant lines represented by two to four independent segregants (Figure S1). This comprehensive collection provides a valuable genetic resource to study the effect of different *Tamlo* mutant alleles on the severity of various wheat diseases. 

**Figure 1:**
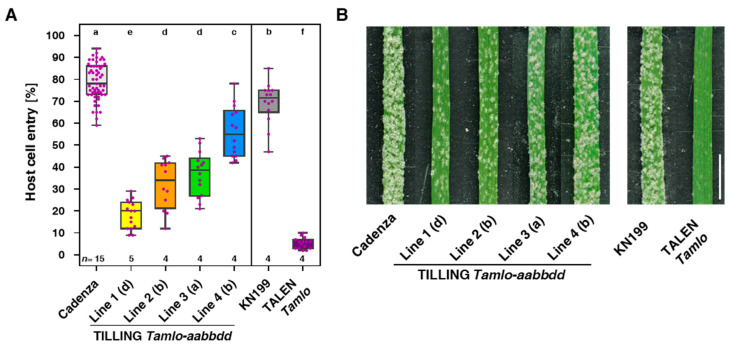
*Blumeria graminis* f.sp. *tritici* (*Bgt*) infection phenotypes of wheat targeted induced local lesions in genomes (TILLING) and transcription-activator-like nuclease (TALEN) *Tamlo* triple-mutants. Primary leaves of 10-day-old TILLING and TALEN *Tamlo* triple-mutants and the corresponding wild-type genotypes, cultivar (cv.) Cadenza and cv. KN199, respectively, were inoculated with conidiophores of *Bgt* isolate JA82. Leaf samples were either harvested at 3 dpi for scoring host cell entry rates (**A**) or photographed at 6 dpi to illustrate macroscopic infection phenotypes (**B**). (**A**) Host cell entry rates for the indicated wheat genotypes were assessed microscopically and are represented as boxplots. Colored dots correspond to the analyzed leaves, which were three to four per genotype and independent biological replicate. At least 200 *Bgt* interaction sites were scored per leaf, i.e., a minimum of 600 inspected interaction sites per genotype and replicate. The number of independent biological replicates is given as *n* above the *x*-axis. Letters above the boxplots denote different significance groups according to statistical analysis (multi-paired analysis of variance (ANOVA) test; *p* < 0.05). (**B**) Macroscopic *Bgt* infection phenotypes of wheat primary leaves of the indicated wheat genotypes at 6 dpi. The white scale bar represents 1 cm.

We challenged the four sets of single-, double- and triple-TILLING mutants and their respective wild-type (cv. Cadenza; spring wheat variety) with *Bgt* by inoculating fixed primary leaves of 10-day-old seedlings with conidiospores and assessed fungal host cell entry rates at 3 dpi as well as the corresponding macroscopic phenotype at 6 dpi. For comparison, we included the previously reported TALEN *Tamlo* triple-mutant [17] together with the respective wild-type genotype (cv. KN199; winter wheat variety). While cv. Cadenza was fully susceptible to *Bgt* with a median host cell entry rate of 78%, representatives of the four TILLING triple-mutant lines showed a gradual decrease in their *Bgt* susceptibility, with line 4 revealing the weakest (55% entry rate) and line 1 the strongest effect (20% entry rate). Lines 2 and 3 exhibited intermediate phenotypes, with 34% and 39% entry rates, respectively (Figure 1A). By contrast, the TALEN triple-mutant was nearly completely resistant to *Bgt* (6% host cell entry) while the respective parental wild-type line, cv. KN199, had an entry rate (72%), somewhat lower than cv. Cadenza (Figure 1A). The microscopically assessed levels of host cell entry rates of all wheat genotypes correlated well with the observed macroscopic infection phenotype (Figure 1). Leaves of cv. Cadenza and cv. KN199 wild-type plants were densely covered by profusely sporulating powdery mildew colonies (Figure 1B). *Bgt* colony density decreased from TILLING triple-mutant line 4 to mutant line 1, indicative of the highest degree of resistance in line 1. In accordance with the low *Bgt* entry rates, the TALEN line was essentially free of macroscopically visible colonies, signifying a very high level of resistance (Figure 1B).

To gain a complete picture regarding the powdery mildew susceptibility/resistance of the TILLING lines, we also assessed the *Bgt* host cell entry rates of the newly selected mutants (summarized in Figure S1). We combined and jointly evaluated the respective data with previous results obtained for some of the single-, double- and triple-mutants of lines 1 and 2 [18]. This comprehensive meta-analysis revealed that none of the single-mutant lines had a host cell entry rate that is statistically different from cv. Cadenza (Figure S2). By contrast, several double-mutant combinations exhibited significantly reduced entry rates, which is indicative of functional redundancy between the three *TaMlo* homoeologs. Notably, in accordance with our earlier report [18], in particular, the *AAbbdd* double-mutant combinations reached host cell entry rates comparable to the respective triple-mutant lines, i.e., not significantly different from the triple-mutant lines according to statistical analysis (Figure S2). In summary, we established a set of TILLING *Tamlo* triple-mutants with differential, allele-specific degrees of powdery mildew resistance, reaching from strong but incomplete resistance in line 1 to rather weak resistance in line 4 (Figure 1).

### 3.2. Tamlo Triple-Mutants Show Unaltered Infection by The Fungal Pathogen Zymoseptoria tritici

**Figure 2:**
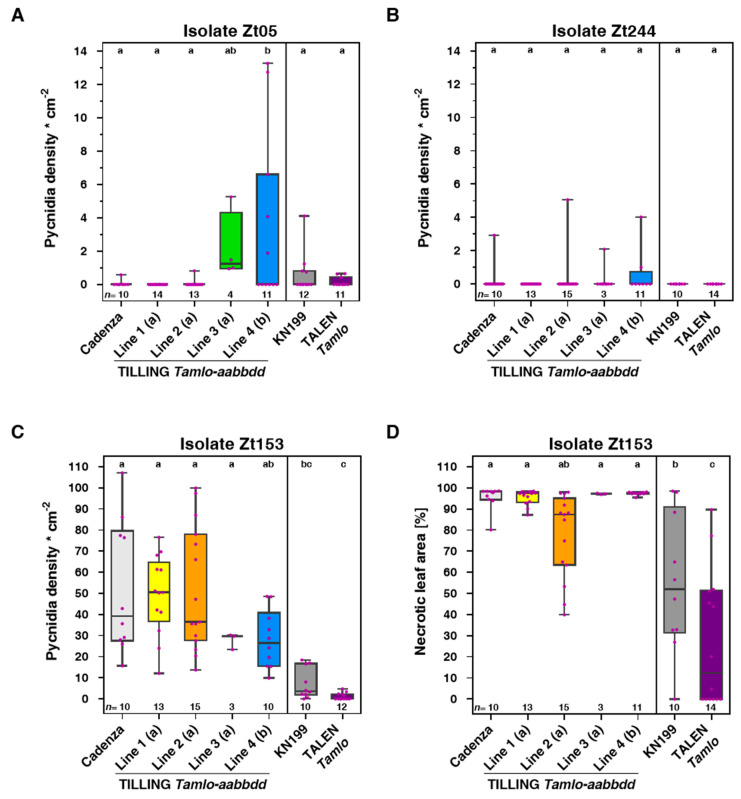
*Tamlo* triple-mutant lines show unaltered infection by the fungal pathogen *Z. tritici*. (**A**–**C**) Boxplots showing the pycnidia density produced by the three *Z. tritici* isolates Zt05 (**A**), Zt244 (**B**) and Zt153 (**C**) at 21 dpi on leaves of the different wheat lines. (**D**) Boxplot of another disease parameter, necrosis, is shown for the isolate Zt153. Necrosis is quantified as % necrotic tissue measured at 21 dpi in a designated leaf area infected with spores. Data shown are from one biological replicate with the number of analyzed leaves given as *n* above the *x*-axis. Letters above the boxplots denote different significance groups according to statistical analysis (multi-paired ANOVA test; *p* < 0.05).

**Figure 3:**
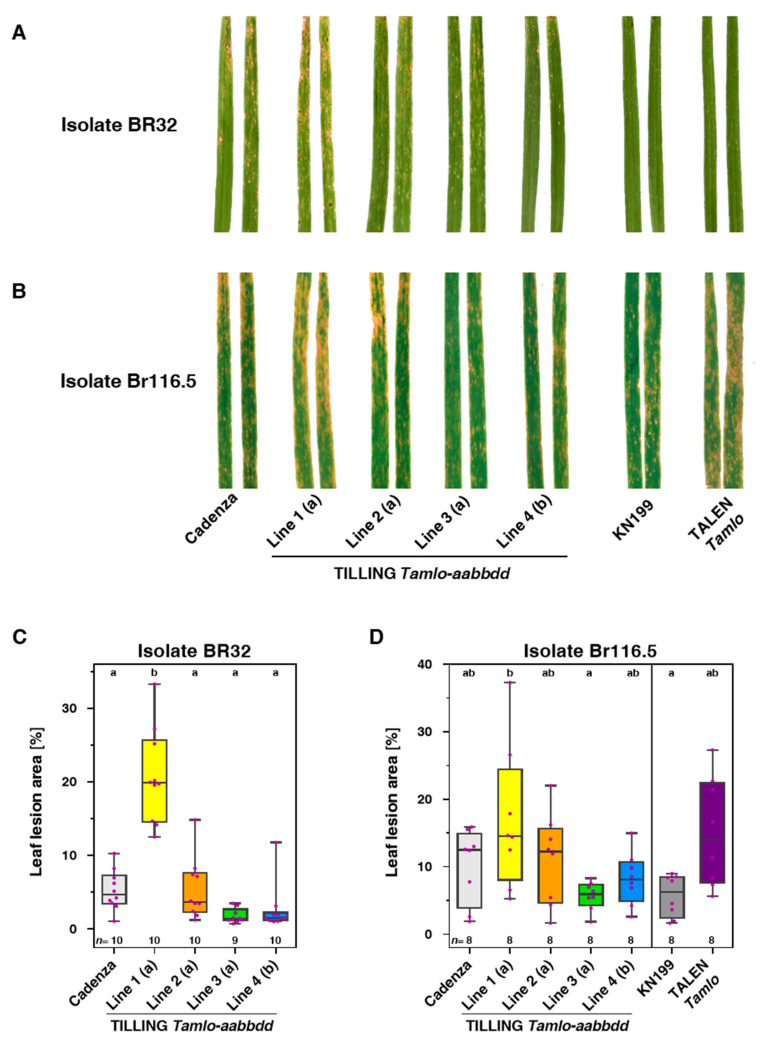
Macroscopic disease symptoms of wheat lines upon challenge with *Magnaporthe oryzae* pv. *Triticum* (*MoT*). (**A**,**B**) Plants of cv. Cadenza and TILLING-lines 1, 2, 3 and 4 and cv. KN199 and the TALEN line, respectively, were inoculated with *MoT* isolate BR32 (**A**) or Br116.5 (**B**). Inoculation was performed on either primary leaves of 14-day-old-plants (**A**) or on secondary leaves of 18-day-old-plants (**B**). Displayed are two representative leaves for each interaction and treatment at 7 dpi. (**C**,**D**) Quantification of diseased leaf areas (blast symptoms) on different wheat genotypes after inoculation with *MoT*. The average diseased leaf areas were calculated based on photographs from eight to ten leaves using the software tool Assess 2.0. Results depicted in (**C**) and (**D**) are from combinations of plant genotypes and fungal isolates corresponding to leaves shown in (**A**) and (**B**), respectively. Leaves of cv. KN199 and the TALEN line showed no visible disease symptoms after inoculation with *MoT* isolate BR32 and were, therefore, omitted in panel (**C**). Data shown in panels (**C**,**D**) are from one biological replicate with the number of analyzed leaves given as *n* above the *x*-axis. Letters above the boxplots denote different significance groups according to statistical analysis (multi-paired ANOVA test; *p* < 0.05).

With the aim to characterize the *Tamlo* mutants further, we next assessed the infection phenotypes of representatives of the four TILLING triple-mutants and the TALEN mutant together with their respective parental lines (cv. Cadenza and cv. KN199, respectively) upon challenge with the hemibiotrophic wheat pathogen *Z. tritici.* To take into account the broad genetic diversity of this pathogen, we used three field isolates of *Z. tritici*—the reference isolate IPO323 (here termed Zt244), originating from The Netherlands, and the isolates Zt05 and Zt153, sampled in Denmark and Germany, respectively. A previous study based on advanced microscopy and transcriptomics described highly different infection phenotypes of the two isolates IPO323 (Zt244) and Zt05 [44]. We here determined the macroscopic infection phenotype and quantified the density of pycnidia and the extent of necrotic leaf area upon challenge with these three isolates. *Z. tritici* isolates Zt05 and Zt244 caused no characteristic disease symptoms on the tested wheat lines (Figure S3), and the density of pycnidia produced by these two isolates was negligible (Figure 2A,B). We thus conclude that cv. Cadenza and cv. KN199 both carry one or more *Septoria tritici blotch* (*Stb*) resistance gene(s) that are effective against isolates Zt05 and Zt244, preventing successful colonization. Isolate Zt153 caused little disease symptoms on cv. KN199 and its derived TALEN *Tamlo* line (Figure S3), which was associated with a moderate density of pycnidia (Figure 2C) and an intermediate level of necrotic leaf area (Figure 3D). In general, the TALEN line appeared to be more resistant to *Z. tritici* than its parental line, though the difference was not visible in terms of asexual reproduction (sporulation). Only the extent of the necrotic leaf area showed a statistically significant difference (Figure 3D). Cv. Cadenza and its derived TILLING *Tamlo* lines were fully susceptible to isolate Zt153, with severe disease symptoms (Figure S3), as evidenced by near-complete leaf necrosis, and a considerable density of pycnidia. However, there was no statistically significant difference between the five wheat genotypes tested regarding these two parameters (Figure 3C,D). We appreciate that field populations of *Z. tritici* can comprise high genetic and phenotypic variation [44]. It is thus possible that other genotypes of *Z. tritici* would induce stronger disease resistance in the wheat lines than observed here. However, based on our experiment with the three tested isolates, we conclude that the *Tamlo* mutations, which condition allele-specific weak to strong disease resistance against the biotrophic powdery mildew pathogen *Bgt* (Figure 1), do not markedly affect the outcome of infection with the hemibiotroph *Z. tritici*.

### 3.3. Tamlo Triple-Mutant Lines Show Allele-Dependent Levels of Enhanced Susceptibility to The Fungal Pathogen Magnaporthe oryzae pv. Triticum

To explore whether wheat *mlo* mutants such as barley *mlo* mutants show altered infection phenotypes in response to blast pathogens, we next assessed the disease phenotypes of the four TILLING and TALEN *Tamlo* triple-mutants together with their respective parental wild-type lines following challenge with the wheat blast pathogen *MoT*. All wheat genotypes were inoculated with either *MoT* isolate BR32 [38] or *MoT* isolate Br116.5 [39], two Brazilian isolates with differential virulence spectrum, and the resulting disease symptoms were macroscopically assessed at 7 dpi (Figure 3). We noted the occurrence of characteristic blast lesions on all wheat genotypes upon challenge with *MoT* isolate Br116.5; however, disease severity varied in a genotype-dependent manner. While cv. Cadenza and the four TILLING lines also exhibited varying degrees of blast lesions upon challenge with *MoT* isolate BR32, cv. KN199 and the TALEN line did not show any disease symptoms in response to this isolate. We thus conclude that KN199 and its derived TALEN line might display genotype-specific (*R* gene-mediated) resistance against isolate BR32. In the following, we thus excluded cv. KN199 and its derived TALEN line from further evaluation regarding this *MoT* isolate.

Macroscopic comparison of disease severity of cv. Cadenza and TILLING lines 1–4 revealed that line 1 was more severely affected by both *MoT* isolates than the wild-type or any of the other TILLING lines (Figure 3A,B). Similar results with both tested *MoT* isolates indicate that this outcome is isolate-independent. Similar to TILLING line 1, the TALEN line showed a higher degree of disease symptoms than its parental wild-type line cv. KN199 upon challenge with *MoT* isolate Br116.5 (Figure 3B). Interestingly, TILLING lines 3 and 4 exhibited slightly fewer disease symptoms than cv. Cadenza with both *MoT* isolates (Figure 3A,B).

To substantiate the results of the macroscopic evaluation, we performed quantitative assessment of the diseased leaf areas using the software APS Assess 2.0 on photographs of infected leaves. Results obtained by this measurement essentially confirmed the visual impressions and revealed a statistically significant difference in the diseased leaf areas between cv. Cadenza and TILLING line 1 in case of the *MoT* isolate BR32 (Figure 3C). The data further in tendency indicate an increase in the leaf lesion area for the TILLING line 1 as compared to cv. Cadenza and of the TALEN mutant in comparison to cv. KN199 in case of the *MoT* isolate BR116.5, although these differences were not significant according to stringent statistical analysis (Figure 3D).

To explore the basis for the different levels of disease susceptibility of the wheat genotypes against *MoT* isolate Br116.5, we microscopically analyzed interaction sites of cv. Cadenza and TILLING line 1 as well as of cv. KN199 and the TALEN line. Therefore, we harvested respective leaf samples at 24 and 96 h post inoculation (hpi) and examined them by consecutive bright-field (to assess the presence of either epiphytical infection structures or bulbous hyphae in epidermal cells) and epifluorescence microscopy (to evaluate the deposition of autofluorescent material). In agreement with our previous studies [42,45], we assigned each interaction site to one of the following five categories (Figure S4): (1) presence of epiphytic fungal infection structures such as conidia or appressoria and absence of autofluorescence; (2) presence of autofluorescent deposits underneath appressoria; (3) whole cell autofluorescence of the attacked epidermal cell; (4) autofluorescence of intact, globularly-shaped mesophyll cells in close contact to attacked epidermal cells; and (5) autofluorescence of collapsed mesophyll cells. In addition, the presence of bulbous infection hyphae within epidermal cells (invasive hyphae) was recorded.

**Figure 4:**
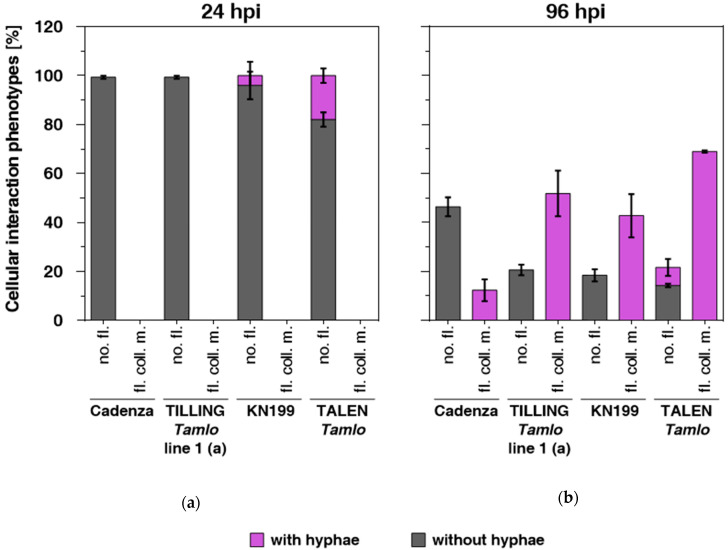
Quantitative assessment of cellular *MoT* interaction phenotypes. Primary leaves of the specified wheat lines were inoculated with *MoT* isolate BR32 or Br 116.5 and harvested at 24 h post inoculation (hpi) (**A**) or 96 hpi (**B**). After removal of leaf pigments, samples were subjected to bright-field and epi-fluorescence microscopy and each fungal infection site was assigned to one out of five categories (see Figure S4). For simplicity, only data of the two most relevant categories are given, which is why values per genotype do not add to 100% (in particular, at 96 hpi). The categories shown are: no fluorescence at the infection site (no. fl.; category 1 in Figure S4) and fluorescence associated with collapsed mesophyll cells (fl. coll. m.; category 5 in Figure S4). The proportion of infection sites at which fungal hyphae in epidermal tissue (invasive hyphae Figure S4) occurred additionally are marked for each category in magenta. At least 100 interaction sites were evaluated per leaf and genotype. Mean and standard deviation were calculated from three (cv. Cadenza and the TILLING line) or two (cv. KN199 and the TALEN line) leaves for each time point.

In the barley/*M. oryzae* interaction, the beforehand mentioned categories are correlated with the successive invasion of the pathogen from epidermal to mesophyll tissue. For simplicity, we focus here on the most relevant categories 1 and 5, with category 5 being an indicator of advanced mesophyll colonization by the pathogen [46,47]. Inspection of leaf samples revealed that, irrespective of the wheat genotype analyzed, there was hardly any autofluorescence detectable underneath appressoria at 24 hpi (corresponding to category 1), and no collapsed, autofluorescent mesophyll cells (category 5) were detectable in any of the interactions. However, cv. KN199 and the TALEN line showed presence of some fungal hyphae in epidermal cells, with the TALEN line permitting more hyphal growth than its respective wild-type (Figure 4A). At 96 hpi, the frequency of interaction sites without autofluorescence decreased to 46% and 18% for cv. Cadenza and cv. KN199, respectively. Concomitantly, we recorded 12% (cv. Cadenza) and 43% (cv. KN199) of sites with collapsed, autofluorescent mesophyll cells (category 5), which specify successful fungal mesophyll invasion. Accordingly, bright-field microscopy revealed that fungal hyphae were present in epidermal cells at all of these infection sites. In contrast to the corresponding wild-type genotypes cv. Cadenza and cv. KN199, TILLING line 1 and the TALEN mutant showed considerably higher proportions of collapsed mesophyll cells at 96 hpi (category 5; 52% and 69%, respectively; Figure 4). These results are indicative of enhanced mesophyll invasion of the pathogen in the *Tamlo* genotypes, which is consistent with the more pronounced disease symptoms observed for both genotypes in comparison to the parental lines (Figure 3). In summary, we conclude that *Tamlo* triple-mutant lines exhibit allele-dependent increased disease susceptibility to *MoT*, with the stronger *Tamlo* mutant alleles present in TILLING line 1 and the TALEN mutant affecting the outcome of the interaction the most.

### 3.4. Tamlo Triple-Mutants Do Not Exhibit Signs of Early Leaf Senescence

**Figure 5:**
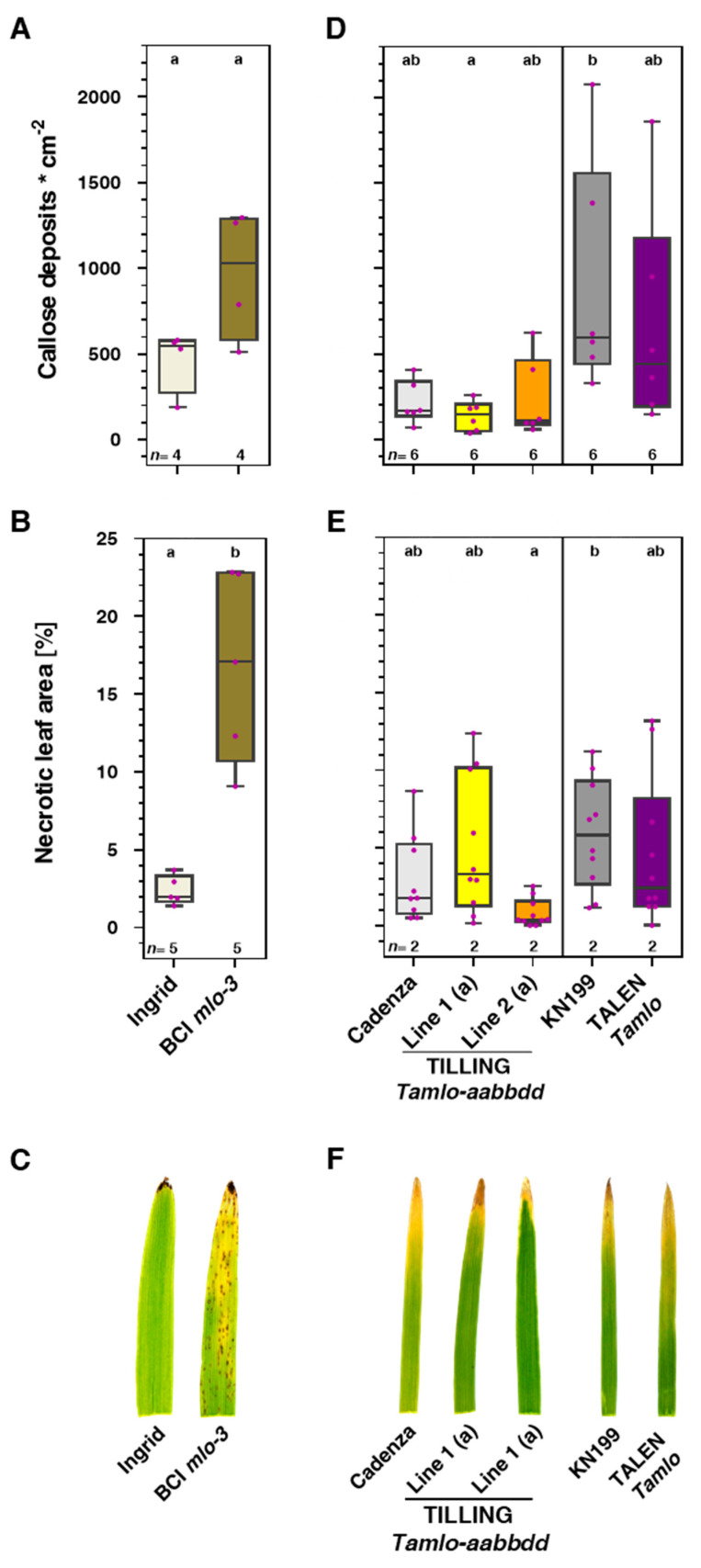
*Tamlo* triple-mutant lines do not exhibit signs of early leaf senescence. (**A**–**C**) Spontaneous callose deposits and leaf necrosis in a barley *mlo* mutant (backcross Ingrid (BCI) *mlo*-3) as compared to the isogenic *Mlo* wild-type (cv. Ingrid). (**A**) Callose deposits in 24-day-old primary barley leaves. Data of individual biological replicates per genotype are displayed as colored dots in the boxplots, and the number of replicates (*n*) is indicated below each boxplot. Each biological replicate represents the mean of three to ten evaluated leaves. Letters above the boxplots denote significantly different groups (*p* < 0.05) according to a regular two-way ANOVA test. (**B**) Quantitative assessment of the extent of macroscopically visible necrosis in primary leaves of 24-day-old barley plants. Data of individual biological replicates per genotype are displayed as colored dots in the boxplots, and the number of replicates (*n*) is indicated below each boxplot. Each biological replicate represents the mean of three to five evaluated leaves. Letters above the boxplots denote different significance groups according to statistical analysis (regular two-way ANOVA test; *p* < 0.05). (**C**) Macroscopic phenotype of primary leaves of 24-day-old barley plants. (**D**–**F**) Spontaneous callose deposits and leaf necrosis in wheat *mlo* mutants as compared to the respective isogenic *Mlo* wild-type (cv. Cadenza or cv. KN199). (**D**) Callose deposits in 31-day-old primary wheat leaves. Data of individual biological replicates per genotype are displayed as colored dots in the boxplots, and the number of replicates (*n*) is indicated below each boxplot. Each biological replicate represents the mean of ten evaluated leaves. Letters above the boxplots denote significantly different groups (*p* < 0.05) according to a one-way ANOVA test with Tukey’s multiple comparisons. (**E**) Quantitative assessment of the extent of macroscopically visible necrosis in primary leaves of 31-day-old wheat plants. The colored dots represent ten evaluated leaves from two independent experiments (*n* = 2; five leaves each). Letters above the boxplots denote different significance groups according to statistical analysis (one-way ANOVA test with Tukey’s multiple comparisons; *p* < 0.05). (**F**) Macroscopic phenotype of primary leaves of 31-day-old wheat plants.

In barley and Arabidopsis, *mlo* mutations are associated with signs of premature leaf senescence and spontaneous callose deposition [12,20,22,23]. To explore whether this is also the case for the *Tamlo* mutants, we grew cv. Cadenza, representatives of the TILLING triple-mutant lines 1 and 2, cv. KN199 and the TALEN line in controlled conditions. At the age of 31 days, we scored the spontaneous occurrence of callose deposits and macroscopically assessed the extent of leaf chlorosis and necrosis in the primary leaves of the plants. As a control, we included a barley wild-type genotype (cv. Ingrid) and a corresponding isogenic *mlo* null mutant (BCI *mlo*-3; [32,33]) in these experiments, which were scored at the age of 24 days. Consistent with a previous report [20], the BCI *mlo*-3 mutant developed substantially more callose deposits than cv. Ingrid (median of 1027 versus 548 deposits cm^−2^; Figure 5A) and showed extensive signs of leaf chlorosis and necrosis, covering approximately 17% of the leaf area (cv. Ingrid: 2%; Figure 5B,C). By contrast, the number of spontaneous callose deposits did not differ significantly between the two tested TILLING lines and cv. Cadenza (median of 143, 107 and 166 deposits cm^−2^, respectively; Figure 5D). The same was true for cv. KN199 and the TALEN line (595 and 442 deposits cm^−2^; Figure 5D), although these two genotypes had a tendency for higher numbers of callose deposits than cv. Cadenza and the two TILLING lines, which likely was caused by an effect of the different genetic backgrounds of these two wheat varieties. None of the five analyzed wheat lines showed obvious signs of early leaf chlorosis and necrosis in our experimental conditions (Figure 5E,F).

## 4. Discussion

We selected and analyzed a set of wheat TILLING-derived *mlo* mutants with various allele combinations (Figure S1). These included novel segregants of two previously characterized *Tamlo* triple-mutant lines (lines 1 and 2) as well as several segregants of two new *Tamlo* triple-mutants (lines 3 and 4). All mutants are based on missense mutations in the three *TaMlo* homoeologs that were previously found to exhibit different levels of residual functionality in a barley single-cell transient expression assay [18]. According to this knowledge, the four TILLING *Tamlo* lines were predicted to reveal different degrees of powdery mildew resistance, which was experimentally confirmed by the results of our bioassays (Figure 1 and Figure S2). The novel segregants corroborated a high level of resistance to *Bgt* in lines 1 and 2, with line 2 being somewhat more susceptible in the current experiments than reported before [18]. As predicted, lines 3 and 4 showed markedly higher *Bgt* entry rates and exhibited more sporulating colonies on the leaf surface (Figure 1 and Figure S2). We noted that intriguingly, in most cases, *Tamlo* double-mutants of the *AAbbdd* genotype showed a similar level of resistance as the corresponding triple-mutants harboring the same allele combination (Figure S2), which is consistent with our previous findings [18]. We conclude that irrespective of the mutated homoeologs a *Tamlo* double-mutant of the *AAbbdd* genotype might be sufficient to obtain almost full protection against *Bgt* infection. We further confirmed the previously reported near-complete resistance of the TALEN *Tamlo* triple-mutant against *Bgt* [17] with our German field isolate JA82 (Figure 1). Altogether, the four TILLING lines, the TALEN line and the corresponding wild-types (cv. Cadenza and cv. KN199) cover a huge spectrum of susceptibility/resistance, ranging from near-complete resistance in the TALEN line to full susceptibility in the wild-types (Figure 1). The wheat *mlo* lines thus recapitulate the situation known from barley, where *mlo* mutant alleles conferring differential levels of powdery mildew resistance have been reported [22,48,49,50]. This set of plants is, therefore, well suited to study the effect of *Tamlo* mutations on the outcome of interactions with other pathogens and the occurrence and strength of putative pleiotropic phenotypes. A similar panel of TILLING-based *mlo* mutants covering a broad range of powdery mildew susceptibility/resistance was recently described for tetraploid durum wheat (*T. turgidum*; [19]).

We tested representatives of the four TILLING lines as well as the TALEN mutant and the respective parental wild-types with two hemibiotrophic pathogens (*Z. tritici* and *M. oryzae*) that differ in life-style from powdery mildew. *Z. tritici* has a long latent phase before it switches to a necrotrophic lifestyle [51]. *M. oryzae* is also classified as a hemibiotrophic pathogen but its initial biotrophic phase is considerably shorter than that of *Z. tritici* [52]. Of the three tested *Z. tritici* isolates, only Zt153 was virulent on the examined wheat genotypes. However, upon challenge with Zt153, the wheat *mlo* mutants did not differ from the respective wild-types regarding the macroscopic infection phenotype (Figure S3) or the density of pycnidia and the extent of the necrotic area (Figure 2). By contrast, TILLING line 1 and the TALEN mutant showed more severe disease symptoms (characteristic blast lesions) upon challenge with *MoT* isolate Br116.5, and TILLING line 1 also upon challenge with *MoT* isolate BR32 (Figure 3). The stronger symptoms correlated with enhanced invasive fungal growth in these two lines (Figure 4). The fact that this outcome was only seen with TILLING line 1 and the TALEN mutant, which exhibit the highest level of powdery mildew resistance (Figure 1), indicates that allele strength may play a decisive role for the establishment of this phenotype. Putative anticorrelation between the degree of powdery mildew resistance and its associated pleiotropic phenotypes depending on *mlo* allele strength has been proposed before [11]. Nonetheless, experiments with additional *Tamlo* mutants will be needed to substantiate these findings further.

The enhanced susceptibility of wheat *mlo* mutants against *MoT* is reminiscent of the enhanced susceptibility of barley *mlo* mutants against *M. oryzae* [26]. More generally, powdery mildew-resistant barley and Arabidopsis *mlo* mutants were reported to have altered infection phenotypes to some additional pathogenic and beneficial microorganisms. Besides their enhanced susceptibility to *M. oryzae*, barley *mlo* mutants were reported to have tissue-dependent enhanced disease resistance against the oomycete pathogen *Phytophthora palmivora* [53] as well as increased sensitivity to toxin-containing culture filtrates of the fungal pathogen *Bipolaris sorokiniana* [54]. By contrast, existing data regarding the fungal pathogens *Fusarium graminearum* and *Ramularia collo-cygni* as well as mycorrhizal fungi are controversial. For spike infections of barley *mlo* mutants with *F. graminearum*, both enhanced susceptibility [55] and unaltered infection phenotypes [56] have been noticed. Similarly, both enhanced leaf susceptibility [57] and unaltered spike infection [56] have been observed for barley *mlo* mutants against *R. collo-cygni*, possibly pointing to tissue- or development-specific differences as in in case of *P. palmivora* where infection was only attenuated in young leaves of an *mlo* mutant [53]. Finally, contrasting outcomes were also seen in experiments studying root colonization by symbiotic fungi. While previously reduced colonization intensity and arbuscule abundance of the mycorrhizal fungus *Funnelliformis mosseae* (former *Glomus mosseae*) was described for a barley *mlo* mutant [58], a recent study did not find such differences for this fungus, but instead reduced colonization with the root-colonizing endophyte *Serendipita indica* [59]. However, another recent report describes reduced colonization by the arbuscular mycorrhizal fungus *Rhizophagus irregularis* in the case of *mlo* mutants in barley, wheat and *Medicago truncatula* [60]. The, in part, contradictory outcomes with barley *mlo* mutants in these studies could be conditioned by experimental differences and environmental factors such as growth conditions, employed microbial isolates and inoculation procedures. The actual methods used for the scoring of infection phenotypes may likewise affect the outcome. Of note, most studies in barley were performed with a single (backcrossed) *mlo* mutant (harboring the *mlo*-5 null allele) and its isogenic parental line (BCI *mlo*-5). The usage of multiple independent *mlo* alleles would greatly improve the credibility of such comparative analyses. Similar to barley *mlo* mutants, Arabidopsis powdery mildew-resistant *mlo* mutants show, in part, altered infection phenotypes to a range of other microorganisms [12,23,61]. However, no clear pattern regarding lifestyle, tissue preference or mode of colonization emerged from these assays. It has been speculated previously that the tendency of *mlo* mutants to undergo spontaneous mesophyll cell death might be generally beneficiary for pathogens that engage in a hemibiotrophic [26] or necrotrophic lifestyle [54,57]. However, the unaltered infection phenotypes of barley, Arabidopsis and wheat *mlo* mutants with some hemibiotrophic/necrotrophic pathogens (for example, our data with *Z. tritici*; Figure 2 and Figure S3) argue against this generalized simplistic explanation. It thus remains to be explored which factor(s) of *mlo* mutants contribute to the modulation of infection phenotypes against a subset of pathogens.

Spontaneous deposition of callose and the occurrence of leaf chlorosis and necrosis have been reported in barley and Arabidopsis *mlo* mutants [12,20]. These phenotypes, which also involve the catabolism of leaf pigments [22,23], reduced photosynthetic performance [23] and the occurrence of mesophyll cell death [62], have been interpreted as a type of premature leaf senescence [22,23]. Notably, similar undesired phenotypes were not seen in the case of tomato and pea *mlo* mutants [24,25]. In the present work, we also failed to detect elevated levels of spontaneous callose deposition or early leaf chlorosis/necrosis in the tested wheat *mlo* mutants (Figure 5). For the TALEN *mlo* line, this outcome contrasts results from our previous study, where we noticed a premature leaf senescence phenotype for this mutant but not for the TILLING *mlo* lines [18]. This seeming discrepancy might be explained by the different developmental stages (seedlings versus mature plants) and/or growth conditions used in the experiments. It is known that the appearance and extent of pleiotropic phenotypes is variable in barley *mlo* mutants and depends on the genetic background and possibly yet unidentified environmental factors [20]. Further experiments will be required to unravel the exact conditions that either promote or protract the development of pleiotropic phenotypes in *mlo* mutants in general and in wheat *mlo* mutants in particular. In summary, we feel that our results are of high relevance for agricultural practice, and we recommend caution regarding the employment of *mlo* wheat genotypes in geographical regions where *MoT* is prevalent.

## Supplementary Materials

The following are available online at https://www.mdpi.com/2073-4425/11/5/517/s1, Figure S1: Summary of all generated wheat TILLING *Tamlo* mutants and their respective allele combinations, Figure S2: *Bgt* infection phenotypes of wheat TILLING *Tamlo* mutants in different allele combinations, Figure S3: Macroscopic infection phenotypes of *Tamlo* mutants treated with the three *Z. tritici* isolates Zt05, Zt153 and Zt244, Figure S4: Categorization of cellular interaction phenotypes of wheat plants challenged with *MoT*.

## Abbreviations

ANOVA	analysis of variance
BCI	backcross Ingrid
*Bgt*	*Blumeria graminis* f.sp. *tritici*
cv.	cultivar
dpi	days post inoculation
hpi	hours post inoculation
Mlo	Mildew locus o
*MoT*	*Magnaporthe oryzae* pv. *Triticum*
TALEN	Transcription activator-like effector nuclease
TILLING	Targeted induced local lesions in genomes
