# Supplementary material for: Evidence for Allele-Specific Levels of Enhanced Susceptibility of Wheat mlo Mutants to the Hemibiotrophic Fungal Pathogen Magnaporthe oryzae pv. Triticum
Source: Genes (Basel). 2020 May 7;11(5):517. doi: 10.3390/genes11050517 (PMC7720134; doi:10.3390/genes11050517)
Supplement: Supplementary file 1 [file genes-11-00517-s001.pdf]

A

Line 1					
Name	*Tamlo-A1* P325L (medium)	*Tamlo-B1* G319R (strong)	*Tamlo-D1* P335L (very strong)	*Mlo* genotype	HCE [%]
Cadenza				*AABBDD*	84
Tamlo-aa BBDD line 1 (a)	X			***aa** BBDD*	80
Tamlo-AA bb DD line 1 (a)		X		*AA **bb** DD*	75
Tamlo-AABB dd line 1 (a)			X	*AABB **dd***	72
Tamlo-aabb DD line 1 (a)	X	X		***aabb** DD*	57
Tamlo-aabb DD line 1 (b)					n.d.
Tamlo-aabb DD line 1 (c)					n.d.
Tamlo-aa BBdd line 1 (a)	X		X	***aa** BB **dd***	78
Tamlo-aa BBdd line 1 (b)					n.d.
Tamlo-AAbb dd line 1 (a)		X	X	*AA **bbdd***	23
Tamlo-AAbb dd line 1 (b)					13
Tamlo-AAbb dd line 1 (c)					26
Tamlo-aabbdd line 1 (a)	X	X	X	***aabbdd***	21
Tamlo-aabbdd line 1 (b)					27
Tamlo-aabbdd line 1 (c)					29
Tamlo-aabbdd line 1 (d)					17
Tamlo-aabbdd line 1 (e)					21

B

Line 2					
Name	*Tamlo-A1* P325L (medium)	*Tamlo-B1* G319R (strong)	*Tamlo-D1* G319R (strong)	*MLO* genotype	HCE [%]
Cadenza				*AABBDD*	84
Tamlo-aa BBDD line 2 (a)	X			***aa** BBDD*	80
Tamlo-AA bb DD line 2 (a)		X		*AA **bb** DD*	75
Tamlo-AABB dd line 2 (a)			X	*AABB **dd***	69
Tamlo-aabb DD line 2 (a)	X	X		***aabb** DD*	39
Tamlo-aa BBdd line 2 (a)	X		X	***aa** BB **dd***	72
Tamlo-AAbb dd line 2 (a)		X	X	*AA **bbdd***	24
Tamlo-AAbb dd line 2 (b)					25
Tamlo-aabbdd line 2 (a)	X	X	X	***aabbdd***	23
Tamlo-aabbdd line 2 (b)					34
Tamlo-aabbdd line 2 (c)					42

C

Line 3					
Name	*Tamlo-A1* P325L (medium)	*Tamlo-B1* G296E (strong)	*Tamlo-D1* P321S (medium)	*MLO* genotype	HCE [%]
Cadenza				*AABBDD*	77
Tamlo-aa BBDD line 3 (a)	X			***aa** BBDD*	76
Tamlo-AA bb DD line 3 (a)		X		*AA **bb** DD*	82
Tamlo-AABB dd line 3 (a)			X	*AABB **dd***	79
Tamlo-aabb DD line 3 (a)	X	X		***aabb** DD*	51
Tamlo-aabb DD line 3 (b)					n.d.
Tamlo-aa BBdd line 3 (a)	X		X	***aa** BB **dd***	41
Tamlo-aa BBdd line 3 (b)					n.d.
Tamlo-aa BBdd line 3 (c)					n.d.
Tamlo-aa BBdd line 3 (d)					n.d.
Tamlo-AAbb dd line 3 (a)		X	X	*AA **bbdd***	42
Tamlo-aabbdd line 3 (a)	X	X	X	***aabbdd***	39
Tamlo-aabbdd line 3 (b)					41
Tamlo-aabbdd line 3 (c)					n.d.

D

Line 4					
Name	*Tamlo-A1* P325L (medium)	*Tamlo-B1* T297I (strong)	*Tamlo-D1* P321S (medium)	*MLO* genotype	HCE [%]
Cadenza				*AABBDD*	78
Tamlo-aa BBDD line 4 (a)	X			***aa** BBDD*	76
Tamlo-AA bb DD line 4 (a)		X		*AA **bb** DD*	83
Tamlo-AABB dd line 4 (a)			X	*AABB **dd***	79
Tamlo-aabb DD line 4 (a)	X	X		***aabb** DD*	66
Tamlo-aabb DD line 4 (b)					n.d.
Tamlo-aa BBdd line 4 (a)	X		X	***aa** BB **dd***	86
Tamlo-AAbb dd line 4 (a)		X	X	*AA **bbdd***	51
Tamlo-AAbb dd line 4 (b)					n.d.
Tamlo-AAbb dd line 4 (c)					n.d.
Tamlo-aabbdd line 4 (a)	X	X	X	***aabbdd***	63
Tamlo-aabbdd line 4 (b)					55
Tamlo-aabbdd line 4 (c)					56

HCE: host cell entry; mean percentage value from several experimental repetitions
n.d.: not determined

Supplemental Figure 1. Summary of all generated wheat TILLING *Tamlo* mutants and their respective allele combinations.

The Figure summarizes features of single-, double-, and triple-mutant combinations of the four TILLING *Tamlo* lines, line 1 (yellow, **A**), line 2 (orange, **B**), line 3 (green, **C**), and line 4 (blue, **D**), generated by stacking of different mutant alleles of the wheat *Mlo* homoeologs *TaMlo-A1*, *TaMlo-B1*, and *TaMlo-D1*. For each of the four lines, the available single-, double- and triple mutants are given (column “Name”) and their respective genotypes are indicated (column “*MLO* genotype”). Each mutant is characterized by distinctive *Tamlo* alleles, leading to particular amino acid substitutions, whose presence is indicated by crosses in the columns “*TaMlo-A1*”, “*TaMlo-B1*”, and “*TaMlo-D1*” and specifically quoted for each line in the same column head (second line). These alleles were assessed previously for their resistance-conferring capacity (medium, strong, or very strong, as given in brackets below the amino acid substitutions; [18]). Note that some mutant combinations are represented by several independent segregants, distinguished by lower-case letters (a, b, c …) following the mutant name. The column HCE indicates the mean host cell entry rate, as experimentally determined with *Bgt* isolate JA82. Data of TILLING *Tamlo* genotypes that are shaded in grey were published before [18].

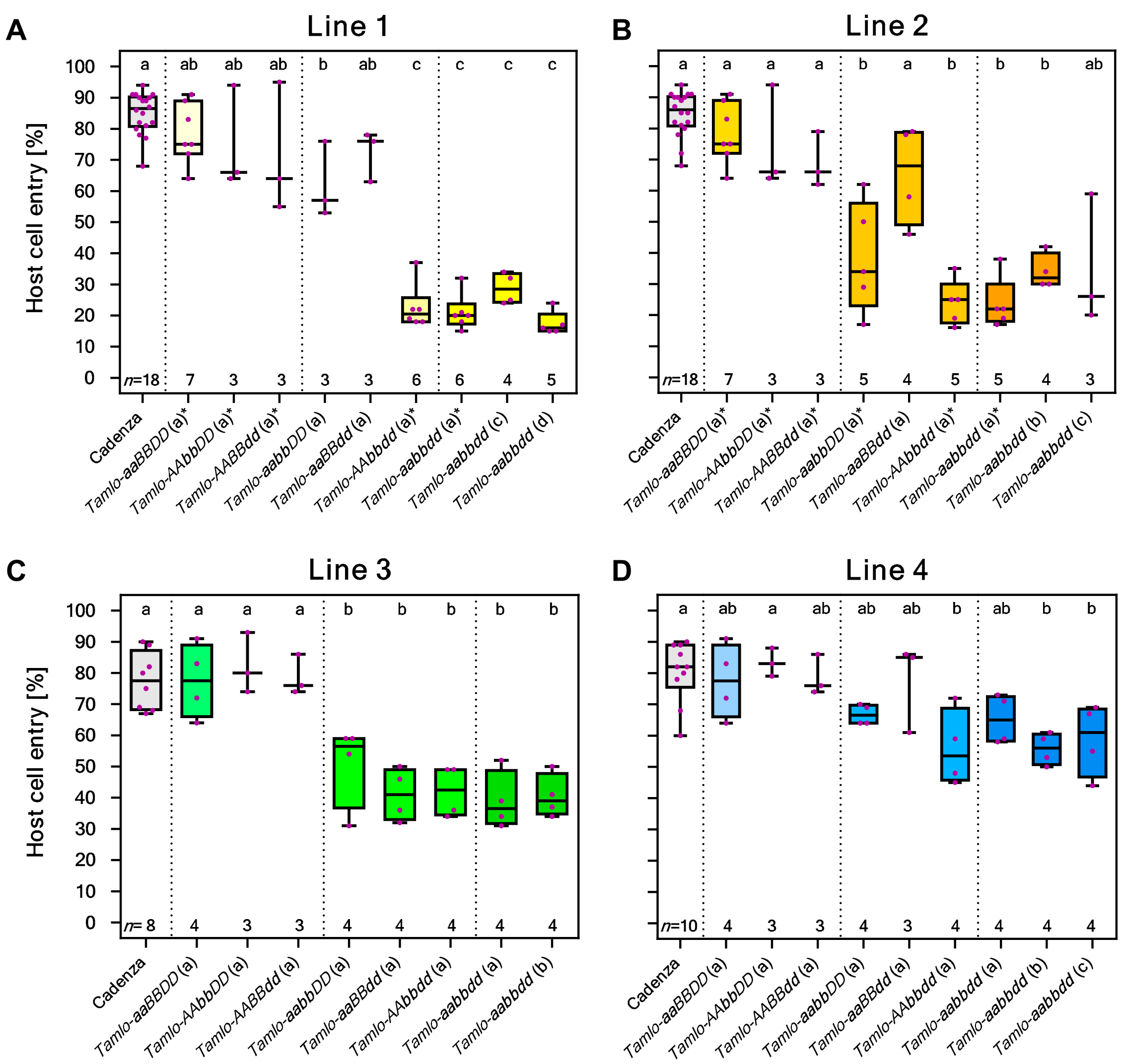

Supplemental Figure 2. *Bgt* infection phenotypes of wheat TILLING *Tamlo* mutants in different allele combinations.

Primary leaves of 10-day-old wheat TILLING *Tamlo* single-, double-, and triple mutants (indicated by their respective genotype) of the four *Tamlo* lines described in Supplemental Figure 1 and the corresponding cv. Cadenza wild-type were fixed on a polycarbonate platform and inoculated with conidiophores of *Bgt* isolate JA82. Host cell entry rates for the indicated wheat genotypes were assessed microscopically at 3 dpi and are represented as boxplots. Data shown relate to line 1 (yellow, **A**), line 2 (orange, **B**), line 3 (green, **C**) and line 4 (blue, **D**). Data of individual biological replicates per genotype are displayed as colored dots, and the total number of biological replicates is given as n above the x-axis. Each biological replicate represents the mean of three or four evaluated leaves per genotype, with at least 200 *Bgt* interaction sites scored per leaf, i.e., a minimum of 600 inspected interaction sites per

genotype and replicate. Letters above the boxplots denote grouping according to a multi-paired ANOVA test ($p<0.05$). Data of TILLING *Tamlo* genotypes marked with an asterisk were published before (Acevedo-Garcia et al., 2017b).

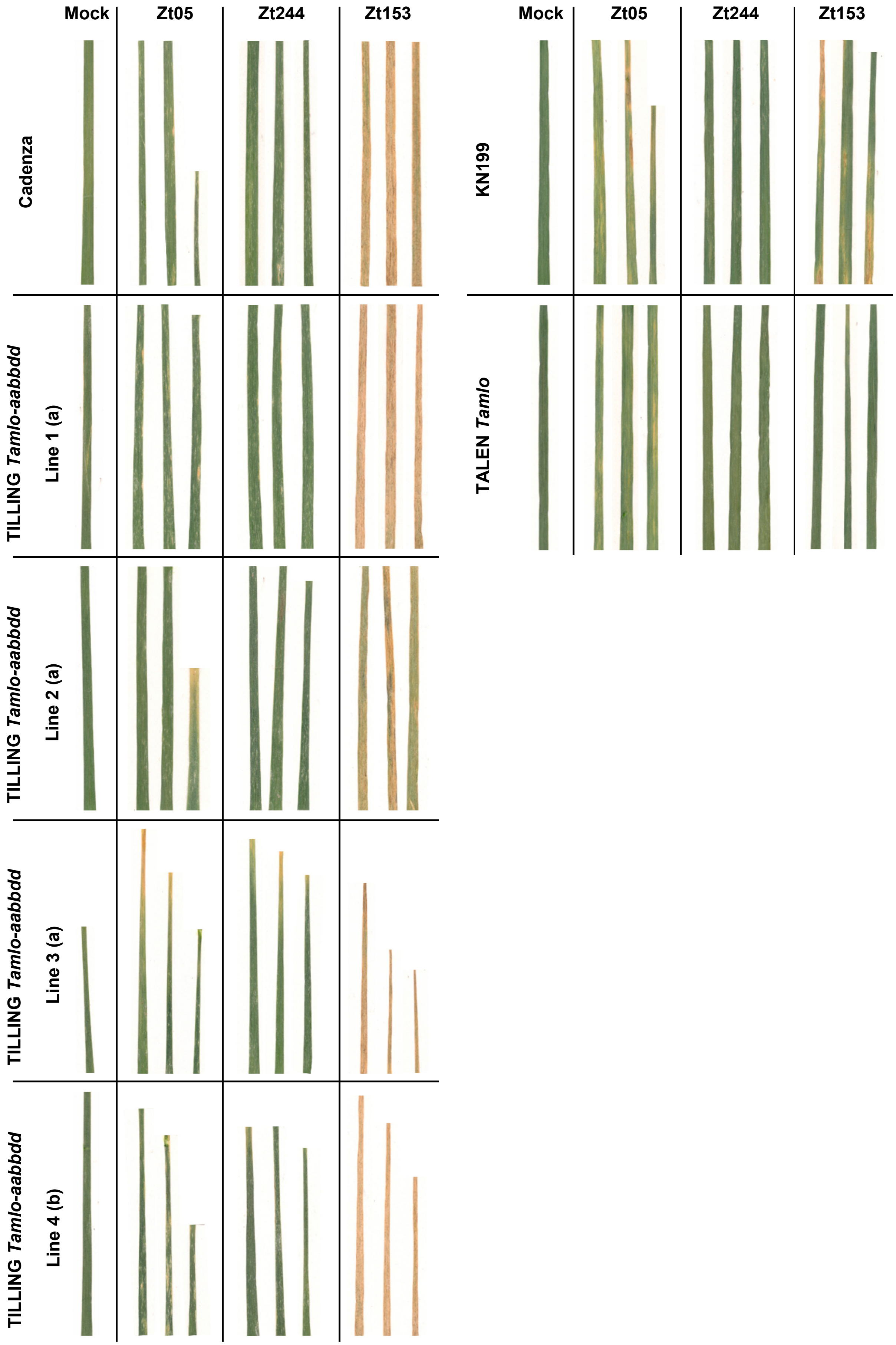

Mock
Zt05
Zt244
Zt153
Cadenza
TILLING *Tamlo-aabbdd*
Line 1 (a)
TILLING *Tamlo-aabbdd*
Line 2 (a)
TILLING *Tamlo-aabbdd*
Line 3 (a)
TILLING *Tamlo-aabbdd*
Line 4 (b)
Mock
Zt05
Zt244
Zt153
KN199
TALEN *Tamlo*

Supplemental Figure 3. Macroscopic infection phenotypes of *Tamlo* mutants treated with the three *Z. tritici* isolates Zt05, Zt153 and Zt244.

Three representative leaves are shown for each plant genotype and fungal isolate at the time point 21 dpi. Disease symptoms are visible as necrosis and pycndia produced by *Z. tritici*.

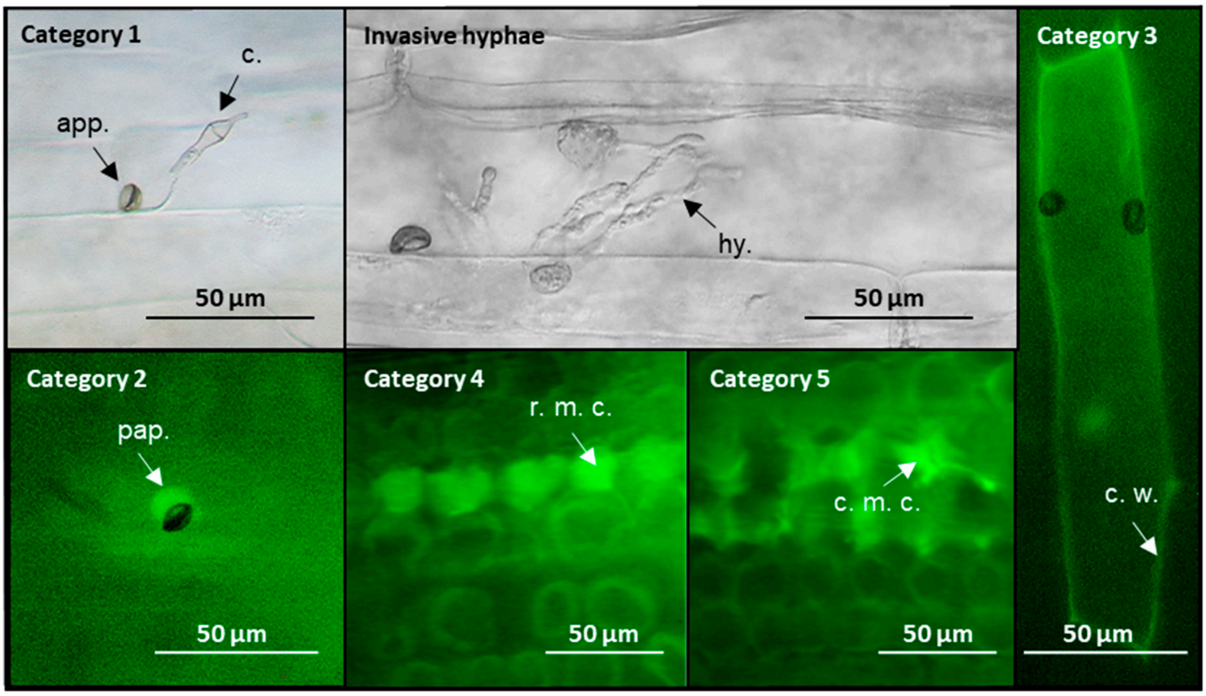

Supplemental Figure 4. Categorization of cellular interaction phenotypes of wheat plants challenged with *MoT*.

Primary leaves of wheat lines infected with *MoT* were harvested at different time points after inoculation. After extraction of leaf pigments, samples were evaluated by bright-field (category 1 and detection of invasive hyphae) or epi-fluorescence microscopy (categories 3 to 5). The micrographs shown illustrate different types of plant responses expressed by cells beneath fungal appressoria. Category 1, cell without any autofluorescence; category 2, local accumulation of autofluorescent material close to the appressorium; category 3, autofluorescence of cell walls of attacked epidermal cell; category 4, autofluorescence of round-shaped mesophyll cells; and category 5, autofluorescence of collapsed mesophyll cells. In addition, the occurrence of hyphae within epidermal cells (invasive hyphae) was recorded. Conidium (c.), appressorium (app.), papilla (pap.), bulbous hyphae (hy.), round-shaped mesophyll cell (r. m. c.), collapsed mesophyll cell (c. m. c.) and cell wall (c. w.) are marked with arrows.
